# Characterization of B cell activation and spatial distribution in the tumor microenvironment of high-grade serous ovarian cancer

**DOI:** 10.1016/j.tranon.2026.103004

**Published:** 2026-08-27

**Authors:** Eleonora Capezzali, Martina Arcieri, Silvia Tonon, Carolina Ricci, Viviana Valeri, Stefano Restaino, Lorenza Driul, Benedetta Gomba, Sara Pregnolato, Alessandro Mangogna, Laura Mariuzzi, Angelica Tulisso, Maria Orsaria, Claudia Andreetta, Carlo Pucillo, Giuseppe Vizzielli, Barbara Frossi

**Affiliations:** a Department of Medicine, University of Udine, Udine, Italy; b Clinic of Obstetrics and Gynecology, "Santa Maria Della Misericordia" University Hospital, Azienda Sanitaria Universitaria Friuli Centrale, Udine, Italy; c Institute of Pathology, "Santa Maria Della Misericordia" University Hospital, Azienda Sanitaria Universitaria Friuli Centrale, Udine, Italy; d Department of Medical Oncology, "Santa Maria Della Misericordia" University Hospital, Azienda Sanitaria Universitaria Friuli Centrale, Udine, Italy

**Keywords:** Ovarian cancer, B cells, Tumor microenvironment, Neoadjuvant chemotherapy, Tumor-infiltrating lymphocytes

## Abstract

**Objective:**

To characterize the distribution and activation status of B cell subsets in tumor tissue and peripheral blood from patients with high-grade serous ovarian cancer (HGSOC) diagnosis, and to assess B cell alterations following neoadjuvant chemotherapy (NACT) in relation to clinical response.

**Methods:**

This translational observational study enrolled patients with FIGO stage III–IV HGSOC from the MICO trial (NCT06272240). Tumor and peripheral blood samples were collected at baseline—during primary debulking surgery (PDS) or diagnostic laparoscopy—and at interval debulking surgery (IDS). Peritoneal biopsies and blood samples were also obtained from healthy controls. Multiparametric flow cytometry and immunohistochemistry were used to characterize B cell phenotype, localization, and dynamics. Clinical data (BRCA status, survival) were included in our analysis.

**Results:**

Among the 30 evaluable patients, B cells accounted for 2–28% of tumor-infiltrating immune cells. Tumor tissues collected from HGSOC patients were enriched in CD19^+^ B lymphocytes, enriched in memory and antibody-secreting cells (ASCs), which were significantly more abundant compared to healthy controls. At baseline, circulating CD71^+^ B cells were significantly increased in HGSOC patients relative to healthy controls. Percentage of infiltrated B cells positive for CD21 was increased after NACT, suggesting functional maturation. Histological analysis showed that, after IDS, patients with better prognosis displayed B-cell clusters in both intratumoral and peritumoral regions; those who experienced early relapse did not.

**Conclusions:**

Preliminary results regarding B-cell distribution and activation in HGSOC indicate dynamic changes that may correlate with clinical outcomes. Spatially organized B cells may serve as favorable immune indicators and biomarkers for treatment stratification.

## Highlights

• Peritoneal HGSOC tissues show enriched CD45+ and CD19+ B cells vs healthy controls.
• Expanded flow cytometry profiling reveals B-cell subsets in HGSOC.
• Flow cytometric analysis suggests a local activation of B lymphocytes.
• NACT shifts tumor-infiltrating B cells toward a mature, activated phenotype.
• NACT response correlates with organized B-cell aggregates over sparse infiltration.

## Introduction

High-grade serous ovarian cancer (HGSOC) is the most common and deadliest subtype of epithelial ovarian cancer, accounting for the majority of advanced-stage diagnoses and cancer-related deaths among women. Despite treatment advances, the five-year survival rate for HGSOC remains below 50%, mainly due to late-stage detection in over 70% of cases and a high recurrence rate after initial treatment [1,2].

Standard treatment of advanced HGSOC consists of either primary debulking surgery (PDS) followed by chemotherapy, or neoadjuvant chemotherapy (NACT) followed by interval debulking surgery (IDS) and subsequent completion of chemotherapy. The treatment strategy is selected based on tumor resectability and the patient’s overall clinical status [[3], [4], [5]]. First-line standard of care typically combines carboplatin and paclitaxel, with maintenance therapy using targeted agents such as anti-angiogenics and PARP inhibitors. In case of relapse, treatment choice is guided by platinum sensitivity and may include non-platinum-based agents [6,7].

Although HGSOC is considered an immunogenic tumor, exhibiting substantial infiltration by immune cells, clinical trials involving immunotherapeutic strategies—including checkpoint inhibitors, CAR-T cells, and cancer vaccines—have shown modest response with little impact on overall survival [8,9]. The tumor microenvironment (TME) in ovarian cancer comprises a complex network of immune and stromal cells, whose interactions may critically modulate disease progression and therapeutic response [10,11].

Although much attention has been given to T lymphocytes, recent research has started to uncover the role of B cells in the ovarian immune TME (TIME). Beyond antibody production, B cells can act as antigen-presenting cells, release regulatory or effector cytokines, and form tertiary lymphoid structures, which may boost antitumor immunity or, conversely, promote immune suppression [12,13]. Nevertheless, their prognostic and functional importance in ovarian cancer remains debated, with conflicting reports describing both positive and negative outcomes depending on B cell phenotype, location, and interaction with other immune components [[14], [15], [16]].

This study focuses on a relatively underexplored aspect of tumor immunology by investigating the distribution and activation status of B cells and their subsets in advanced HGSOC, both in tumor tissue and peripheral blood, and suggesting that B cell profiles may provide insights into treatment response. Using multiparametric flow cytometry and histological analysis, we preliminarily investigated changes induced by NACT and explored potential correlations between B cell profiles, tumor response, and clinical outcomes.

## Materials and methods

### Study design and patients

This study reports preliminary findings from the MICO (MICroenvironment of Ovarian Cancer) project (ClinicalTrials.gov Identifier: NCT06272240), a translational observational study conducted at the Azienda Ospedaliera Universitaria Santa Maria della Misericordia in Udine, Italy. Patient enrolment began in December 2023 and is currently ongoing. Women aged 18–80 years with newly diagnosed, histologically confirmed high-grade serous ovarian cancer (HGSOC) at FIGO stage III–IV were prospectively enrolled after providing written informed consent. Exclusion criteria included ongoing immunosuppressive treatment, other malignancies under active treatment within the previous six months, a body mass index (BMI) > 30, congenital or acquired immunodeficiencies, or absence of surgical indication. The study protocol was approved by the local ethics committee (Protocol number: 38242, dated October 20, 2023).

All patients underwent staging laparoscopy to assess tumor load and determine a predictive index (PI) score [17,18]. Based on PI scores, patients were assigned to either primary debulking surgery (PDS) or neoadjuvant chemotherapy (NACT) followed by interval debulking surgery (IDS). Peritoneal biopsies were collected during diagnostic surgical procedures (t = 0) and after NACT (t=IDS) and were immediately sent fresh to the Department of Medicine at the University of Udine for processing. Peripheral blood samples were obtained at the time of diagnosis and prior to IDS.

The healthy control group included women undergoing risk-reducing bilateral salpingo-oophorectomy and peritoneal biopsies due to genetic predisposition to ovarian cancer, in the absence of active disease [19].

Technical aspects of the protocol have been thoroughly described in our previously publication [20].

### Blood samples

Peripheral blood samples were collected in tubes containing anticoagulants, specifically sodium citrate and EDTA, prior to surgical procedures. Peripheral blood mononuclear cells (PBMCs) were isolated using Ficoll® Paque Plus (GE Healthcare Pharmacia, #17-1440-03) and SepMateTM 50 mL Tubes (Stem Cell Technologies, #154500) following manufacturer’s protocol. Cell pellet was resuspended in Bambanker™ cryopreservation medium (NIPPON Genetics Europe GmbH, #BB02) and stored at −80°C.

### Peritoneal biopsies

Tumor peritoneal biopsies from HGSOC patients were rinsed with phosphate-buffered saline (PBS), transferred to Petri dishes containing AdDF+++ medium (Advanced DMEM/F12 containing L-Glutamine, 10 mM HEPES and Penicillin-Streptomycin), and mechanically dissociated into small fragments. Tissues were then enzymatically digested with collagenase type IV in the presence of Y-27632 and Primocin at 37 °C for 1 hour. All reagents were from Thermo-Fisher. The resulting cell suspension was filtered to remove debris, centrifuged, and resuspended in fresh AdDF+++. Viable cells were counted using Trypan Blue exclusion. For cryopreservation, cells were resuspended in Bambanker™ cryopreservation medium and stored at –80 °C.

### Flow cytometry

To analyze composition of immune cell infiltration, we first assessed CD45^+^ immune cells and subsequently focused on B cell subpopulations through multiparametric flow cytometry. A previously validated antibody panel was used to identify B cell subsets including naïve (CD19^+^ CD24^+/low^ CD38^+/low^ CD27^-^ IgD^+^), memory (CD19^+^ CD24^+^ CD38^-^ CD27^+^ IgD^-^), antibody secreting (ASCs) (CD19^+^ CD24^-^ CD38^++^ CD27^+^ IgD^-^), and Double Negative (DN) (CD19^+^ CD27^-^ IgD^-^) B cells [20]. All antibodies were purchased from Thermo Fisher Scientific or BioLegend.

### Histological analysis

Formalin-fixed, paraffin-embedded tumor tissue sections were stained with antibodies against CD20 and cytokeratin 7 (CK7). B cell localization was assessed by light microscopy and categorized as intratumoral, peritumoral, or organized in lymphoid aggregates. Immune cell density in the tumor microenvironment was assessed using a semiquantitative four-tier scoring system adapted from previously published tumor-infiltrating lymphocyte grading methods [21,22]. Scores were assigned as follows: grade 0 (absent), grade 1 (mild/moderate focal or mild multifocal infiltrate), grade 2 (marked focal, moderate/marked multifocal or mild diffuse infiltrate), and grade 3 (moderate/marked diffuse infiltrate. Histological evaluation was performed independently and in a blind manner by two experienced pathologists.

### Clinical data and statistical analysis

Clinical variables included demographic characteristics, 2014 FIGO stage [23], surgical details, BRCA mutation status (germline and somatic), homologous recombination deficiency (HRD) status, response to chemotherapy, and disease recurrence. Statistical analyses were performed using Prism software. Group comparisons were conducted using non-parametric tests (Mann–Whitney U or Wilcoxon matched-pairs tests). A p-value < 0.05 was considered statistically significant. Progression-free survival (PFS) was evaluated using the Kaplan–Meier method and compared between responder and relapsing patients following NACT by means of the log-rank test. Statistical significance was defined as a two-sided p-value < 0.05. Statistical analysis was conducted using JASP software.

## Results

### Patient characteristics

Of the 50 patient-derived samples collected, 30 met the inclusion criteria for final analysis. The median age at diagnosis was 70.4 years. Among these patients, 13 underwent primary debulking surgery (PDS), while 17 received neoadjuvant chemotherapy (NACT) followed by interval debulking surgery (IDS). BRCA mutations were detected in 9 patients (29.8%), and homologous recombination deficiency (HRD) was observed in 23.3% of cases. During the follow-up period, e8 patients died and 8 experienced disease relapses. In this preliminary study, we analyzed 38 samples at baseline (t = 0): 13 from patients who underwent PDS, 17 from diagnostic laparoscopy, 8 from healthy controls. Additionally, 6-paired samples collected both at t = 0 and t=IDS were evaluated. Patient characteristics are summarized in Table 1.

**Table 1:** Clinical and molecular characteristics of enrolled patients.

	Age at diagnosis	FIGO Stage	Mutational status	Type of surgery	CRS	Maintenance therapy	Relapse	Death
**OV2**	68	III	gBRCA2	IDS	2	Bevacizumab	+	+
**OV3**	55	III	gBRCA1	PDS		Olaparib		
**OV4**	72	III	sBRCA1	IDS	2	Olaparib	+	+
**OV6**	64	III	sBRCA2	PDS		Olaparib		
**OV7**	60	IV	sBRCA1	IDS	2	Olaparib	+	
**OV8**	82	IV	WT	PDS		Niraparib	+	+
**OV9**	50	IV	WT	IDS	2	Bevacizumab		
**OV10**	83	III	gBRCA1	IDS	2	Olaparib	+	+
**OV11**	70	IV	HRD^+^	IDS	1	Olaparib + Bevacizumab		
**OV12**	58	III	HRD^+^	PDS		Olaparib + Bevacizumab	+	+
**OV13**	66	III	WT	IDS	1	Niraparib		
**OV17**	64	IV	gBRCA2	IDS	2	Olaparib		
**OV21**	70	IV	WT	IDS	3	Olaparib		
**OV22**	73	III	gBRCA1	PDS		Olaparib		
**OV25**	75	III	WT	IDS	3	Niraparib		
**OV27**	82	III	HRD^+^	PDS		No		
**OV28**	80	IV	Not suitable	IDS		No		+
**OV31**	66	III	sBRCA1	PDS		Olaparib		
**OV32**	76	III	WT	PDS		Bevacizumab		
**OV33**	72	IV	WT	IDS	2	Olaparib		
**OV34**	79	III	WT	PDS		Niraparib	+	
**OV35**	67	III	WT	PDS		Olaparib		
**OV37**	84	III	WT	IDS		No		+
**OV38**	77	III	WT	PDS		Niraparib		
**OV40**	77	III	WT	IDS		No		+
**OV41**	57	IV	HRD^+^	IDS	2	Bevacizumab + Olaparib		
**OV44**	78	III	WT	IDS		Bevacizumab		
**OV46**	68	III	WT	PDS		Bevacizumab		
**OV50**	69	III	WT	IDS		Niraparib	+	

WT: wild type; sBRCA1: somatic BRCA1 mutation; sBRCA2: somatic BRCA2 mutation; gBRCA1: germinal BRCA1 mutation; gBRCA2: germinal BRCA2 mutation; HRD^+^: homologous recombination deficiency; PDS: primary debulking surgery; IDS: interval debulking surgery; CRS: Chemotherapy Response Score (from 1 to 3).

### Tumor-infiltrating B cells at baseline (t = 0)

**Fig. 1:**
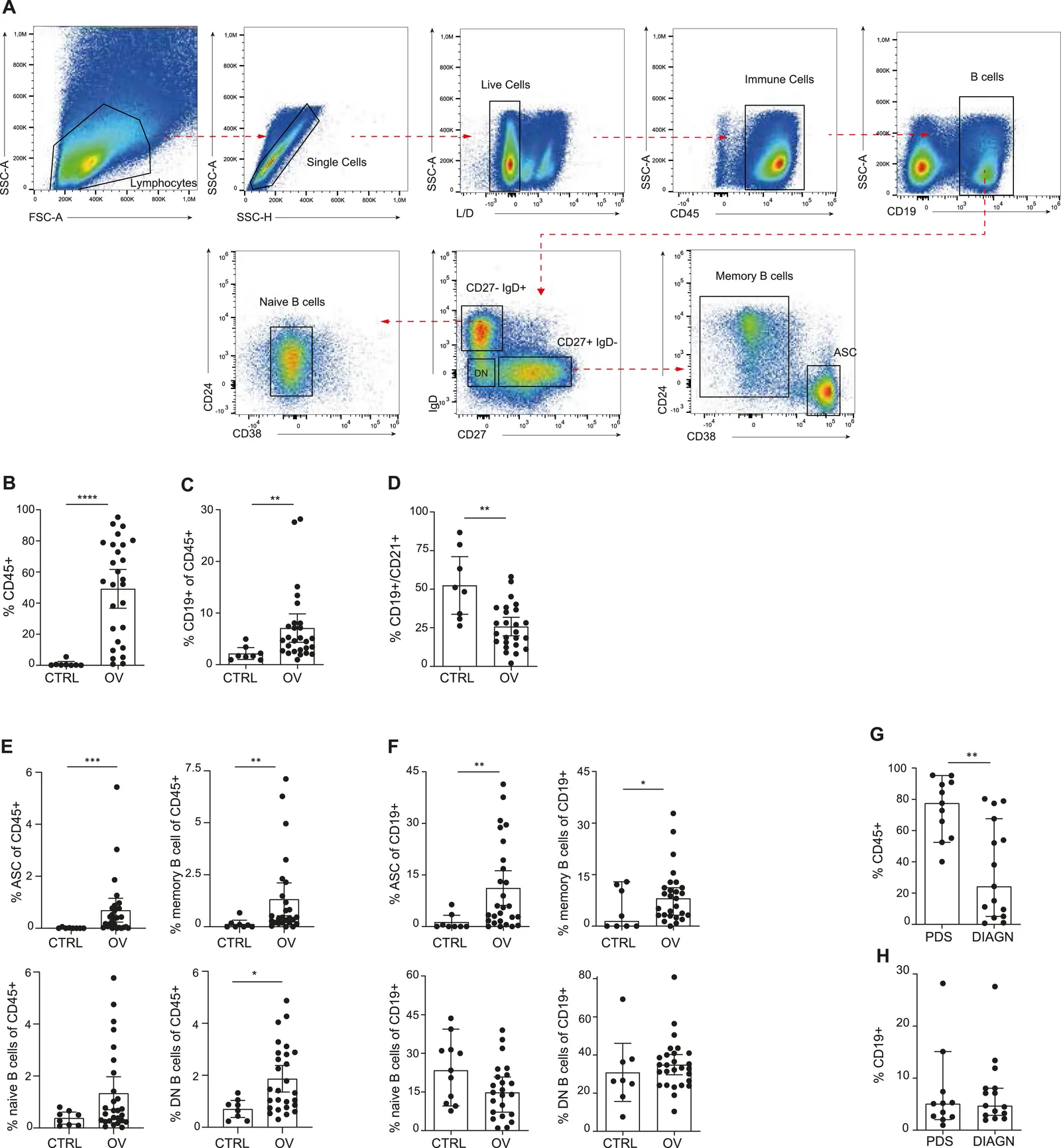
Tumor infiltrating B cells and their subpopulations in peritoneal tissue of healthy controls and HGSOC patients (undergoing PDS or NACT). (A) Representative gating strategy used to identify B cell subpopulations in tumor tissue; (B-D) CTRL vs OV: (B) % of CD45^+^ infiltrate; (C) % of B cells; (D) % of CD19^+^CD21^+^ B cells; (E) antibody-secreting (ASCs), naive, memory, double negative (DN) B cells on CD45^+^ immune infiltrate; (F) antibody-secreting (ASCs), memory, naive, double negative (DN) B cells on CD19^+^ B cells; (G-H) PDS vs NACT: (G) CD45^+^ infiltrate; (H) B cells. CTRL: healthy controls; OV: HGSOC patients; PDS: primary debulking surgery; DIAGN: diagnostic surgery. Mann Whitney statistical test, P value < 0.05 = * P value < 0.01= **, P value < 0.001 = ***.

Fig. 1A shows the gating strategy employed to identify the B subpopulations within the TME of a representative HGSOC patient.

Flow cytometric analysis revealed elevated infiltration of CD45^+^ cells (Fig. 1B) and CD19^+^ B lymphocytes (Fig. 1C) in peritoneal tumor tissue from HGSOC patients compared to healthy controls. B cells accounted for 2–28% of CD45^+^ cells, with marked inter-patients’ heterogeneity. Moreover, CD21—a marker of B cell maturation—resulted significantly decreased in patients with respect to healthy controls (Fig. 1D), together with other differences in the distribution of B cell subsets. Specifically, HGSOC patients exhibited significantly higher frequencies of antibody secreting cells (ASCs), memory B cells and CD27^-^/IgD^-^ double negative B cells, compared to healthy controls. In contrast, the proportion of naïve B cells was similar between the two cohorts, although HGSOC samples showed high dispersion in these populations within CD45^±^ compartment (Fig. 1E). A similar trend was observed if expressed as percentage of the total B cell population, where ASCs and memory B cell fractions were more abundant in patients than in controls (Fig. 1F).

Patients undergoing PDS exhibited a higher density of total immune cells (median CD45^+^: 80%) compared to those undergoing only diagnostic laparoscopy (median CD45^+^: 35%), although, the relative proportion of B cells remained comparable between the two groups (Fig. 1G, H).

We analyzed B cell subpopulations by stratifying patients according to key clinical and molecular characteristics including FIGO stage (III vs. IV), mutational status (WT, *BRCA^mut^,* HRD^+^) and clinical outcome (responder vs. relapsing) to identify patterns in immune infiltration and B cell distribution that might correlate with disease relapses or therapeutic response. As shown in Supplementary Figures S1–3, our analysis revealed considerable inter-patient variability. While some trends began to emerge, they did not reach statistical significance.

### Circulating B cell profiles at baseline (t = 0)

**Fig. 2:**
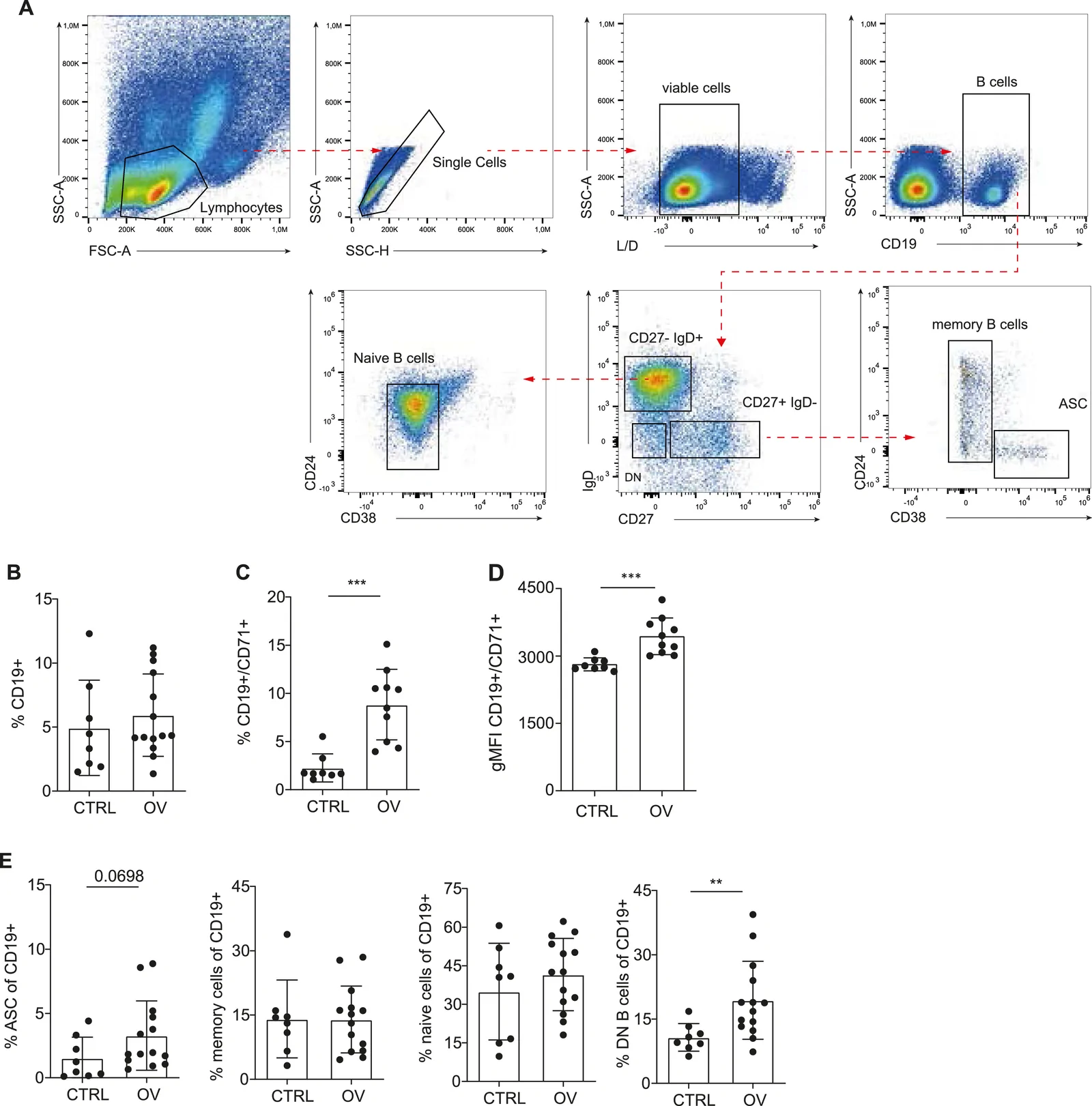
Circulating B cells and their subpopulations from PBMCs of healthy controls vs HGSOC patients*.* (A) Representative gating strategy of a sample; (B) B cells; (C) CD19^+^CD71^+^ B cells (D) gMFI of CD71 marker of CD19+ cells; (E) antibody-secreting (ASCs), memory, naive, double negative (DN) B cells. CTRL: healthy controls; OV: HGSOC patients. Mann Whitney statistical test, P value < 0.01= **, P value < 0.001 = ***.

To characterize the circulating B cell phenotype in HGSOC patients compared to healthy controls, we applied a specific gating strategy (Fig. 2A) to identify total B cells and their subpopulations.

Peripheral blood analysis revealed comparable overall B cell percentages between patients and healthy donors (Fig. 2B). However, a significant increase in both the number of CD19/CD71 double positive B cells (Fig. 2C) and the intensity of the CD71^+^ expression (Fig. 2D) was observed in patients, suggesting B cell systemic activation. Further subset analysis revealed a slight increase in ASCs and a robust and significative increment of circulating CD27^-^/IgD^-^ DN B cells in HGSOC patients compared to controls (Fig. 2D).

### Dynamic changes after NACT: flow cytometric and histological analysis

**Fig. 3:**
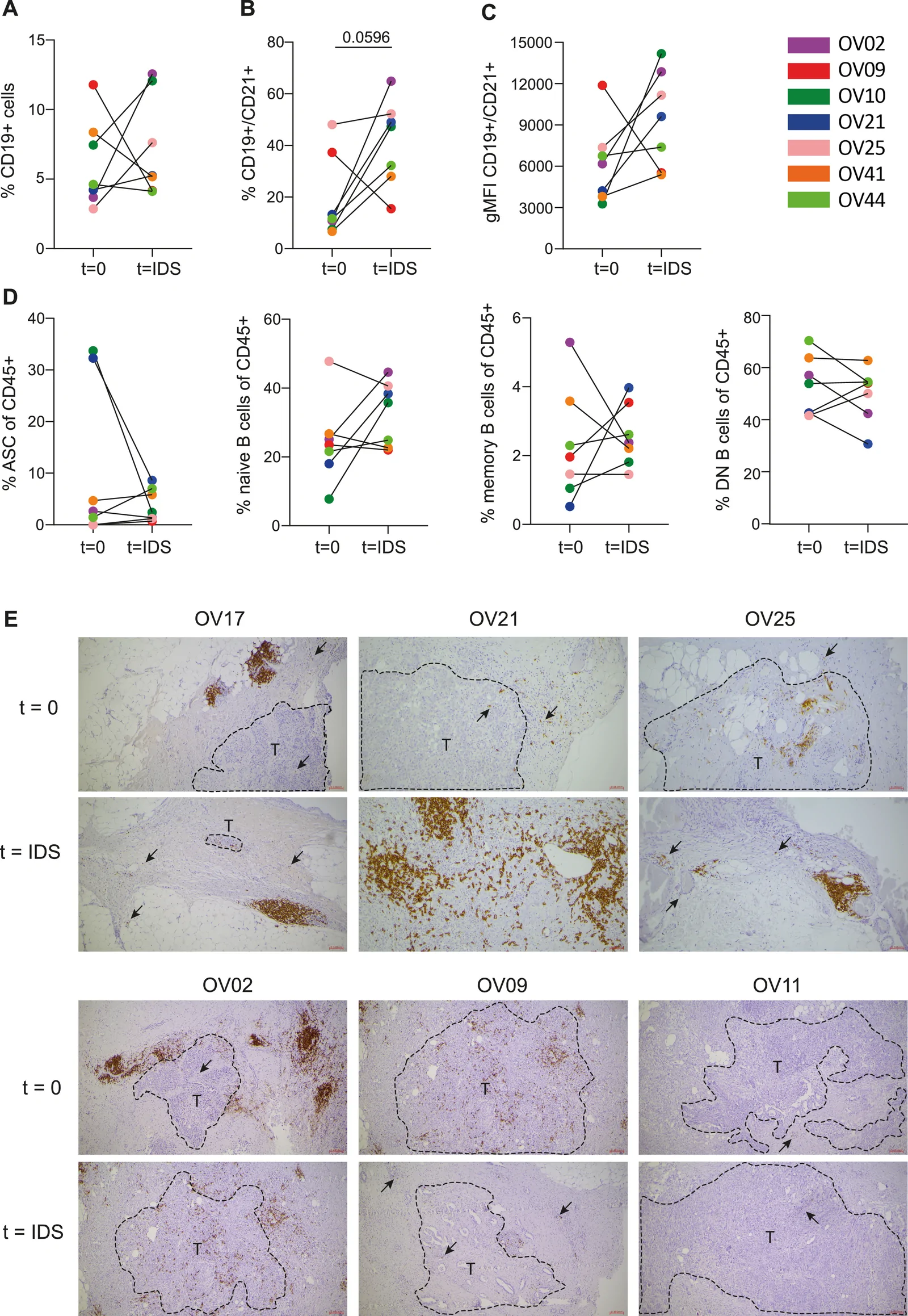
Tumor infiltrating B cells and their subpopulations in peritoneal tissue of HGSOC patients before (t = 0) and after (t = IDS) 3 cycles of NACT. (A) B cells; (B) CD21^+^ B cells; (C) antibody-secreting (ASCs), naive, memory, double negative (DN) B cells. (D) Representative IHC images of B cells of 3 responders (upper box) and relapsing (bottom box) HGSOC patients before and after 3 cycles of NACT. Arrows; sparse CD20^+^ cells; dotted lines: margins of tumoral area (T). All images were taken at 5x magnification; 200 μm scale bar. Paired *t*-test statistical test.

Paired pre- and post-NACT (t = 0 *vs* t=IDS) analyses (n = 7) revealed interpatient variability in the modulation of B cell subsets (Fig. 3A-B; each color corresponds to a single patient).

At t=IDS, CD19^+^ B cells did not show a significant increase in tumor infiltration (Fig. 3A), however, a trend toward higher percentages of CD21 positive B cells was observed (Fig. 3B). This may suggest an expansion of B cells undergoing functional maturation. Meanwhile, ASCs levels either decreased or remained stable, while naïve, memory, and CD27⁻/IgD⁻ DN subsets showed no substantial fluctuations (Fig. 3C).

In peripheral blood, the overall percentage of circulating B lymphocytes decreased following NACT, although distribution of B cell subpopulations remained unchanged.

Finally, we performed a histological analysis of B cell localization in peritoneal biopsies by CD20 staining. Fig. 3D shows the presence of both large B cell cluster and scattered B cells (indicated by arrows) throughout the tissue. The latter are localized in peritumoral as well as intratumoral (T) areas, which are indicated by dotted lines (Table 2).

**Table 2:** Evaluation of B cell localization within the tumor before the initiation of NACT and at IDS. Intratumoral, peritumoral and lymphoid aggregation in 3 responder (upper box) and 3 relapsing (bottom box) HGSOC patients before and after 3 cycles of NACT.

	B cells		
RESPONDING	Intratumoral	Peritumoral	Lymphoid aggregates
**OV17 t=0**	-/(+ focally)	-	+++
**OV17 t=IDS**	+/++	-/(+ focally)	Less organized structures
**OV21 t=0**	-	-/+	Poorly organized structures
**OV21 t=IDS**	++	++	Well organized structures
**OV25 t=0**	-	-	Poorly organized structures
**OV25 t=IDS**	-	-	Poorly organized structures
			
			
	**B cells**		
**RELAPSING**	**Intratumoral**	**Peritumoral**	**Lymphoid aggregates**
**OV02 t=0**	-/(+ focally)	+	+++
**OV02 t= IDS**	+/++	+/++	+++
**OV09 t=0**	-	-/+ (focally)	Barely noticeable structures
**OV09 t=IDS**	-	-/+ (focally)	Barely noticeable structures
**OV11 t=0**	-	-	No structures
**OV11 t=IDS**	-	-	No structures

Notably, histological evaluation revealed that patients who achieved a complete or partial response to NACT (OV17, OV21, OV25) and remained disease-free during the study follow-up tended to display preserved or increased B-cell aggregates within both peritumoral and intratumoral regions (Fig. 3D and Table 2), frequently organized as nodular lymphoid aggregates. Consistent with this observation, CD4^+^ and CD8^+^ T-cells were found in close spatial association with CD20^+^ B-cell aggregates (Suppl. Fig. S4). In contrast, such lymphoid architecture was less frequent or lacking in relapsing patients (OV2, OV9, OV11), who instead exhibited sparse B-cell infiltration or a complete absence of organized aggregates (Table 2 and Fig. 3D). Finally, Kaplan–Meier analysis demonstrated significantly longer progression free survival in responder patients compared with relapsing patients (log-rank test: χ² = 5.052, p = 0.025). No disease progression or relapse events were observed among responders during follow-up, whereas relapsing patients exhibited a median PFS of 3 months (Suppl. Fig. S5).

## Discussion

The role of B lymphocytes in the tumor microenvironment (TME) of several cancer types is receiving increasing attention. Current research is focused on elucidating the diverse functions of these cells and the mechanisms that influence their activity [[24], [25], [26]]. In particular, their role in high-grade serous ovarian cancer (HGSOC) remains only partially understood. While T-cell-mediated immunity has traditionally dominated the field of ovarian cancer immunology, emerging evidence suggests that B cells might exert diverse–and sometimes opposing–effects within the TME. This preliminary study provides a detailed immunophenotypic characterization of tumor-infiltrating and circulating B-cell subsets in patients with advanced-stage HGSOC, with aim of exploring their potential biological and clinical relevance. To our knowledge, this is the first study to employ an expanded flow cytometry panel specifically designed to dissect B-cell subsets in this patient population.

Consistent with previous reports [27,28], we observed substantial interpatient heterogeneity in B-cell frequencies. Moreover, our observations indicate that B cells are consistently present within the HGSOC immune microenvironment, contributing to its overall complexity. Flow cytometric analysis revealed increased infiltration of CD19^+^ B lymphocytes in tumor tissue, particularly ASCs and memory B cells, which were significantly more abundant than in healthy controls. These findings may be compatible with local activation of B lymphocytes although functional studies would be required to confirm their effector role.

In peripheral blood, the observed elevated expression of the proliferation marker CD71 among CD19^+^ B cells at baseline is consistent with systemic B-cell activation in HGSOC. This was accompanied by a modest increase in ASCs and a significant expansion of CD27^-^/IgD^-^ double negative (DN) B cell, a subset often associated with chronic inflammation or immune dysregulation [29].

Following NACT (t=IDS), tumor-infiltrating B cells exhibited increased CD21 expression. Since CD21 is a key component of B cell receptor (BCR) complex [30], this shift may suggest a process of functional maturation or reprogramming[32]. However, although high CD21 expression on B cells has been associated with improved survival in other malignancies [31,32], its presence alone does not necessarily indicate functional activation or effective anti-tumor activity [33]. In the immunosuppressive TME B cells may also remain functionally inert or even acquire regulatory properties. Notably, one patient (OV9), who experienced early relapse and subsequently died, exhibited an atypical post-treatment decrease in CD21^+^ B cells, raising the possibility of a prognostic relevance of this marker.

A key aspect of our preliminary study was the comparison between patients undergoing primary debulking surgery (PDS) and those receiving NACT followed by IDS. At baseline (t = 0), patients selected for PDS — typically indicative of lower tumor burden — showed a higher density of CD45⁺ immune infiltrates, despite similar proportion of B cells, suggesting that overall immune cell abundance may reflect anti-tumor immune response.

Interestingly, the spatial organization of B cells, rather than their mere presence, appeared to be associated with clinical outcomes in our cohort. Patients who did not experience relapse showed nodular lymphoid aggregates composed of CD20^+^ B cells in close association with CD4^+^ and CD8^+^ T lymphocytes, whereas such structures were uncommon or absent in relapsing patients. Although their functional role cannot be inferred from histological observations alone, the coordinated arrangement of B and T cells may reflect the establishment of a locally organized antitumor immune response. The preferential presence of these aggregates in responder patients raises the possibility of an association between lymphoid organization and treatment efficacy [34,35]. Indeed, the presence of spatially organized lymphoid structures has been associated with enhanced local immune surveillance and improved prognosis in several malignancies, including non-small cell lung cancer (NSCLC) and melanoma [36].

Collectively, our data support the notion that B cells in HGSOC exhibit phenotypic diversity and may be dynamically influenced by therapy. Rather than identifying a single dominant B-cell subset associated with disease outcome, our findings point to coordinated changes across multiple B-cell populations, including ASCs, memory B cells and phenotypically activated subsets, together with evidence of lymphoid organization within the TME. The observed enrichment of ASCs, together with changes in activation-associated markers and lymphoid organization, is compatible with the hypothesis that B cells may contribute to effective antitumor immunity, in line with findings by Kroeger et al. [28]. However, the dual roles of B cells—encompassing both pro- and anti-tumor potentials—must be carefully considered. Regulatory B cells — especially those found in ascitic fluid — are known to secrete immunosuppressive cytokines such as IL-10 and TGF-β, and have been associated with tumor-promoting mechanisms, including immune evasion and angiogenesis [37]. Although these subsets were not directly assessed in our study, their potential role warrants further investigation.

Several limitations should be noted. First, the relatively small sample size, particularly in the paired pre- and post-NACT analyses, limits the generalizability of our findings and underscores the need for validation in larger, prospective cohorts. Second, although our flow cytometric panels allowed for high-resolution immunophenotyping, functional assays (e.g., cytokine secretion, antigen presentation) were not performed and it would be necessary to directly assess the effector functions of the identified B cell subsets. Finally, although histological evaluation suggested associations between B-cell architecture and clinical outcomes, these observations remain descriptive and would benefit from integration with spatial transcriptomics or multiplex immunohistochemistry.

## Clinical and translational implications

This study highlights the potential of B cells, including ASCs and spatially organized clusters as immunological biomarkers and therapeutic targets in HGSOC. Although preliminary and requiring validation in larger cohorts, our observations suggest that the assessment of nodular lymphoid aggregates on routine histopathological sections could represent a simple and cost-effective tool to support patient stratification. If confirmed, these structures may serve as a useful indicator of the tumor immune microenvironment and help identify patients more likely to respond to treatment.

Moreover, our findings suggest that, in the future, specific B-cell signatures could be incorporated into future predictive models for patient stratification, particularly in the context of NACT or immunotherapy trials. Furthermore, immunomodulatory strategies aimed at enhancing B-cell activation or promoting the formation of organized lymphoid structures could complement standard chemotherapy or immune checkpoint blockade. Continued efforts to elucidate broader immunological networks in HGSOC are essential for validating these hypotheses and assessing their potential for clinical practice.

In conclusion, our findings indicate that B cells within the HGSOC microenvironment are not only present but also exhibit distinct phenotypic characteristics that may hold potential clinical relevance. The dynamic interplay between tumor and immune cells, therapeutic interventions and clinical outcomes underscore a promising avenue for future research focused on integrating humoral immunity into precision immuno-oncology strategies for ovarian cancer.

## Funding

The work was performed thanks to the support of the Departmental Strategic Plan (PSD) of the University of Udine-Interdepartmental Project on Healthy Ageing (2020-25) and thanks to the support of AIRC (AIRC_IG_2022_ID_27884_PUCILLO), and a donation from Loto OdV Onlus.

## CRediT authorship contribution statement

**Eleonora Capezzali:** Writing – original draft, Data curation, Conceptualization. **Martina Arcieri:** Writing – original draft, Methodology, Data curation, Conceptualization. **Silvia Tonon:** Formal analysis, Data curation. **Carolina Ricci:** Formal analysis, Data curation. **Viviana Valeri:** Formal analysis, Data curation. **Stefano Restaino:** Validation, Supervision. **Lorenza Driul:** Validation, Supervision. **Benedetta Gomba:** Writing – original draft, Data curation. **Sara Pregnolato:** Data curation. **Alessandro Mangogna:** Data curation. **Laura Mariuzzi:** Writing – review & editing, Formal analysis, Conceptualization. **Angelica Tulisso:** Formal analysis, Data curation. **Maria Orsaria:** Writing – review & editing, Conceptualization. **Claudia Andreetta:** Writing – original draft, Validation. **Carlo Pucillo:** Writing – review & editing. **Giuseppe Vizzielli:** Writing – review & editing. **Barbara Frossi:** Writing – review & editing, Conceptualization.

## Declaration of competing interest

The authors declare that they have no known competing financial interests or personal relationships that could have appeared to influence the work reported in this paper.

## Appendix Supplementary materials

Image, application 1

## References

[1] Lheureux S., Gourley C., Vergote I., Oza A.M. (2019). Epithelial ovarian cancer. Lancet.

[2] Sambasivan S. (2022). Epithelial ovarian cancer: review article. Cancer Treat. Res. Commun..

[3] Fagotti A., Ferrandina M.G., Vizzielli G., Pasciuto T., Fanfani F., Gallotta V. (2020). Randomized trial of primary debulking surgery versus neoadjuvant chemotherapy for advanced epithelial ovarian cancer (SCORPION-NCT01461850). Int. J. Gynecol. Cancer..

[4] Mahner S., Heitz F., Salehi S., Reuss A., Guyon F., Du Bois A. (2025). TRUST: Trial of radical upfront surgical therapy in advanced ovarian cancer (ENGOT ov33/AGO‐OVAR OP7). J. Clin. Oncol..

[5] González-Martín A., Harter P., Leary A., Lorusso D., Miller R.E., Pothuri B. (2023). Newly diagnosed and relapsed epithelial ovarian cancer: ESMO clinical practice guideline for diagnosis, treatment and follow-up. Ann. Oncol..

[6] Arcieri M., Tius V., Andreetta C., Restaino S., Biasioli A., Poletto E. (2024). How BRCA and homologous recombination deficiency change therapeutic strategies in ovarian cancer: a review of literature. Front. Oncol..

[7] Arcieri M., Andreetta C., Tius V., Zapelloni G., Titone F., Restaino S. (2025). Molecular biology as a driver in therapeutic choices for ovarian cancer. Int. J. Gynecol. Cancer.

[8] Zhang L., Conejo-Garcia J.R., Katsaros D., Gimotty P.A., Massobrio M., Regnani G. (2003). Intratumoral T cells, recurrence, and survival in epithelial ovarian cancer. N. Engl. J. Med..

[9] Luvero D., Plotti F., Aloisia A., Montera R., Terranova C., De Cicco Nardone C. (2019). Ovarian cancer relapse: from the latest scientific evidence to the best practice. Crit. Rev. Oncol. Hematol..

[10] Cesari E., Ciucci A., Pieraccioli M., Caggiano C., Nero C., Bonvissuto D. (2023). Dual inhibition of CDK12 and CDK13 uncovers actionable vulnerabilities in patient-derived ovarian cancer organoids. J. Exp. Clin. Cancer Res..

[11] Nero C., Vizzielli G., Lorusso D., Cesari E., Daniele G., Loverro M. (2021). Patient-derived organoids and high grade serous ovarian cancer: from disease modeling to personalized medicine. J. Exp. Clin. Cancer Res..

[12] Ghisoni E., Morotti M., Sarivalasis A., Grimm A.J., Kandalaft L., Laniti D.D. (2024). Immunotherapy for ovarian cancer: towards a tailored immunophenotype-based approach. Nat. Rev. Clin. Oncol..

[13] Siminiak N., Czepczyński R., Zaborowski M.P., Iżycki D. (2022). Immunotherapy in ovarian cancer. Arch. Immunol. Ther. Exp..

[14] Nielsen J.S., Sahota R.A., Milne K., Kost S.E., Nesslinger N.J., Watson P.H. (2012). CD20+ tumor-infiltrating lymphocytes have an atypical CD27− memory phenotype and together with CD8+ T cells promote favorable prognosis in ovarian cancer. Clin. Cancer Res..

[15] Laumont C.M., Nelson B.H. (2023). B cells in the tumor microenvironment: multi-faceted organizers, regulators, and effectors of anti-tumor immunity. Cancer Cell.

[16] LeBien T.W., Tedder T.F. (2008). B lymphocytes: how they develop and function. Blood.

[17] Fagotti A., Ferrandina G., Fanfani F., Ercoli A., Lorusso D., Rossi M. (2006). A laparoscopy-based score to predict surgical outcome in patients with advanced ovarian carcinoma: a pilot study. Ann. Surg. Oncol..

[18] Petrillo M., Vizzielli G., Fanfani F., Gallotta V., Cosentino F., Chiantera V. (2015). Definition of a dynamic laparoscopic model for the prediction of incomplete cytoreduction in advanced epithelial ovarian cancer: proof of a concept. Gynecol. Oncol..

[19] Marchetti C., Arcieri M., Vertechy L., Ergasti R., Russo G., Zannoni G.F. (2022). Risk reducing surgery with peritoneal staging in BRCA1-2 mutation carriers. A prospective study. Eur. J. Surg. Oncol..

[20] Arcieri M., Capezzali E., Restaino S., Pregnolato S., Mariuzzi L., Mangogna A. (2025). Study of the role of the tumor microenvironment in ovarian cancer (MICO): a prospective monocentric trial. Cancer Rep..

[21] Maibach F., Sadozai H., Seyed Jafari S.M., Hunger R.E., Schenk M. (2020). Tumor-infiltrating lymphocytes and their prognostic value in cutaneous melanoma. Front. Immunol..

[22] Hendry S., Salgado R., Gevaert T., Russell P.A., John T., Thapa B. (2017). Assessing tumor-infiltrating lymphocytes in solid tumors: a practical review for pathologists and proposal for a standardized method from the international immuno-oncology biomarkers working group: part 2: TILs in melanoma, gastrointestinal tract carcinomas, non–small cell lung carcinoma and mesothelioma, endometrial and ovarian carcinomas, squamous cell carcinoma of the head and neck, genitourinary carcinomas, and primary brain tumors. Adv. Anat. Pathol..

[23] Mutch D.G., Prat J. (2014). 2014 FIGO staging for ovarian, fallopian tube and peritoneal cancer. Gynecol. Oncol..

[24] Mion F., Vetrano S., Tonon S., Valeri V., Piontini A., Burocchi A. (2017). Reciprocal influence of B cells and tumor macro and microenvironments in the *ApcMin/+* model of colorectal cancer. Oncoimmunology.

[25] Downs-Canner S.M., Meier J., Vincent B.G., Serody J.S. (2022). B cell function in the tumor microenvironment. Annu. Rev. Immunol..

[26] Martinis E., Tonon S., Colamatteo A., La Cava A., Matarese G., Pucillo C.E.M. (2025). B cell immunometabolism in health and disease. Nat. Immunol..

[27] Ning F., Cole C.B., Annunziata C.M. (2021). Driving immune responses in the ovarian tumor microenvironment. Front. Oncol..

[28] Kroeger D.R., Milne K., Nelson B.H. (2016). Tumor-infiltrating plasma cells are associated with tertiary lymphoid structures, cytolytic T-cell responses, and superior prognosis in ovarian cancer. Clin. Cancer Res..

[29] Sacks D., Baxter B., Campbell B.C.V., Carpenter J.S., Cognard C., Dippel D. (2018). Multisociety consensus quality improvement revised consensus statement for endovascular therapy of acute ischemic stroke. Int. J. Stroke.

[30] Sanz I., Wei C., Jenks S.A., Cashman K.S., Tipton C., Woodruff M.C. (2019). Challenges and opportunities for consistent classification of human B cell and plasma cell populations. Front. Immunol..

[31] Tanimoto K., Yakushijin Y., Fujiwara H., Otsuka M., Ohshima K., Sugita A. (2009). Clinical significance of co-expression of CD21 and LFA-1 in B-cell lymphoma. Int. J. Hematol..

[32] Nichols E.M., Jones R., Watson R., Pepper C.J., Fegan C., Marchbank K.J. (2015). A CD21 low phenotype, with no evidence of autoantibodies to complement proteins, is consistent with a poor prognosis in CLL. Oncotarget.

[33] Martinis E., Tonon S., Valeri V., Colamatteo A., Ricci C., Trevisan C. (2026). Immunophenotypic skewing of B cells toward IgD^−^CD27^−^IgG^+^ subtype and metabolic attenuation in colorectal cancer. Sci. Rep..

[34] Nelson B.H. (2010). CD20+ B cells: the other tumor-infiltrating lymphocytes. J. Immunol..

[35] Nielsen J.S., Nelson B.H. (2012). Tumor-infiltrating B cells and T cells. Oncoimmunology.

[36] Cabrita R., Lauss M., Sanna A., Donia M., Skaarup Larsen M., Mitra S. (2020). Tertiary lymphoid structures improve immunotherapy and survival in melanoma. Nature.

[37] Wei X., Jin Y., Tian Y., Zhang H., Wu J., Lu W. (2016). Regulatory B cells contribute to the impaired antitumor immunity in ovarian cancer patients. Tumor Biol..

