# Supplementary material for: Characterization of B cell activation and spatial distribution in the tumor microenvironment of high-grade serous ovarian cancer
Source: Transl Oncol. 2026 Aug 27;72:103004. doi: 10.1016/j.tranon.2026.103004 (PMC13544323; doi:10.1016/j.tranon.2026.103004)
Supplement: Supplementary file 1 [file mmc1.docx]

**Supplementary Figures**

**Figure S1.** Tumor-infiltrating immune cells, B lymphocytes, and B cell subpopulations in peritoneal tissue of HGSOC patients at different FIGO stages (III vs IV). Flow cytometry analysis was performed on peritoneal samples collected at the time of diagnostic surgery. Data is expressed as percentages of viable cells (for CD45 marker), of CD45^+^ immune cells (for CD19 marker), or CD19⁺ cells (for all the other markers). (**A**) CD45^+^ total immune cells; (**B**) CD19^+^ B lymphocytes; (**C**) CD19⁺CD21⁺ B cells; (**D**) gMFI CD19⁺CD21⁺ B cells; and (**E**) antibody-secreting cells (ASCs), double negative (DN), memory, and naïve B cell subsets. Statistical analyses were performed with Mann–Whitney U test (III n=18-20, IV n=7).

**Figure S2.** Tumor-infiltrating immune cells, B lymphocytes, and B cell subpopulations in peritoneal tissue of HGSOC patients with different mutational status (WT, BRCA^mut^, HRD^+^). Flow cytometric analysis was performed on peritoneal samples collected at the time of diagnostic surgery. Data is expressed as percentages of viable cells (for CD45 marker), of CD45^+^ immune cells (for CD19 marker), or CD19⁺ cells (for all the other markers). (**A**) CD45^+^ total immune cells; (**B**) CD19^+^ B lymphocytes; (**C**) CD19⁺CD21⁺ B cells; (**D**) gMFI CD19⁺CD21⁺ B cells; and (**E**) antibody-secreting cells (ASCs), double negative (DN), memory, and naïve B cell subsets. Statistical analyses were performed with Mann–Whitney U test (WT n=13, BRCA^mut^ n=6-8, HRD^+^ n=6).

**Figure S3.** Tumor-infiltrating immune cells, B lymphocytes, and B cell subpopulations in peritoneal tissue of HGSOC patients with divergent clinical outcomes. Flow cytometric analysis was performed on peritoneal samples collected at the time of diagnostic surgery. Data is expressed as percentages of viable cells (for CD45 marker), of CD45^+^ immune cells (for CD19 marker), or CD19⁺ cells (for all the other markers). (**A**) CD45^+^ total immune cells; (**B**) CD19^+^ B lymphocytes; (**C**) CD19⁺CD21⁺ B cells; (**D**) gMFI CD19⁺CD21⁺ B cells; and (**E**) antibody-secreting cells (ASCs), double-negative (DN), memory, and naïve B cell subsets. Statistical analyses were performed with Mann–Whitney U test (responder n=17-19, relapsing n=8).

***Figure S4.*** *Co-localization of B and T lymphocytes in peritoneal tissue of HGSOC patients*. Immunohistochemistry for CD20, CD4 and CD8 in the biopsy of one representative HGSOC patient (OV17) shows strong co-localization of T and B cells in lymphoid aggregates. Majority of lymphocytes are B cells, some are CD4+ T cells while CD8+ T cells are scarce. Co-localization is suggestive of a local organized immune response.

***Figure S5.*** *Progression-free survival in relapsing versus responder patients***.**

Progression-free survival (PFS) was estimated using the Kaplan–Meier method and compared between relapsing (OV2, OV9, OV11) and responder (OV17, OV21, OV25) patients (pts) using the log-rank test.
